# Double Pinned Perpendicular-Magnetic-Tunnel-Junction Spin-Valve Providing Multi-level Resistance States

**DOI:** 10.1038/s41598-019-48311-0

**Published:** 2019-08-15

**Authors:** Jin-Young Choi, Hansol Jun, Kei Ashiba, Jong-Ung Baek, Tae-Hun Shim, Jea-Gun Park

**Affiliations:** 1 MRAM Center, Department of Electronics and Computer Engineering, Hanyang University, Seoul, 04763 Republic of Korea; 2 MRAM Center, Department of Nanoscale Semiconductor Engineering, Hanyang University, Seoul, 04763 Republic of Korea; 3 Wafer Engineering Department, SUMCO CORPORATION, 1-52 Kubara, Imari, Saga 849-4256 Japan

**Keywords:** Electrical and electronic engineering, Electronic and spintronic devices

## Abstract

A new design for high density integration greater than gigabits of perpendicular-magnetic-tunnel-junction (p-MTJ) spin-valve, called the double pinned (i.e., bottom and top pinned structures) p-MTJ spin-valve achieved a multi-level memory-cell operation exhibiting four-level resistances. Three key magnetic properties, the anisotropy exchange field (*H*_*ex*_) of the bottom pinned structure, the coercivity (*H*_c_) of the double free-layer, and the *H*_c_ of the top pinned structure mainly determined four-level resistances producing tunneling-magnetoresistance (TMR) ratios of 152.6%, 33.6%, and 166.5%. The three key-design concepts are: i) the bottom pinned structure with a sufficiently large *H*_*ex*_ to avoid a write-error, ii) the *H*_c_ of the double free-layer (i.e., ~0.1 kOe) much less than the *H*_c_ of the top pinned structure (i.e., ~1.0 kOe), and iii) the top pinned structure providing different electron spin directions.

## Introduction

Perpendicular spin-transfer-torque magnetic-random access memory (p-STT MRAM), which consists of a perpendicular magnetic tunneling junction (p-MTJ) spin-valve and a selective device, has attracted great research interest because of its possibility in various applications. Recently, in particular, the p-STT MRAM cells have been utilized as embedded memory in system-on-chip for mobile and internet-of-things applications^1–5^, a stand-alone memory as a solution to the dynamic-random-access-memory (DRAM) scaling limitations below the 10-nm node^6^, and spin-neuron and synaptic devices for deep learning^7–9^. The p-STT MRAM has many advantages over current memory devices, such as non-volatility, fast read/write speed (~10 ns), extremely low power consumption (<1 pJ/bit), high write endurance (>10^12^), and scalability^10–15^.

**Figure 1:**
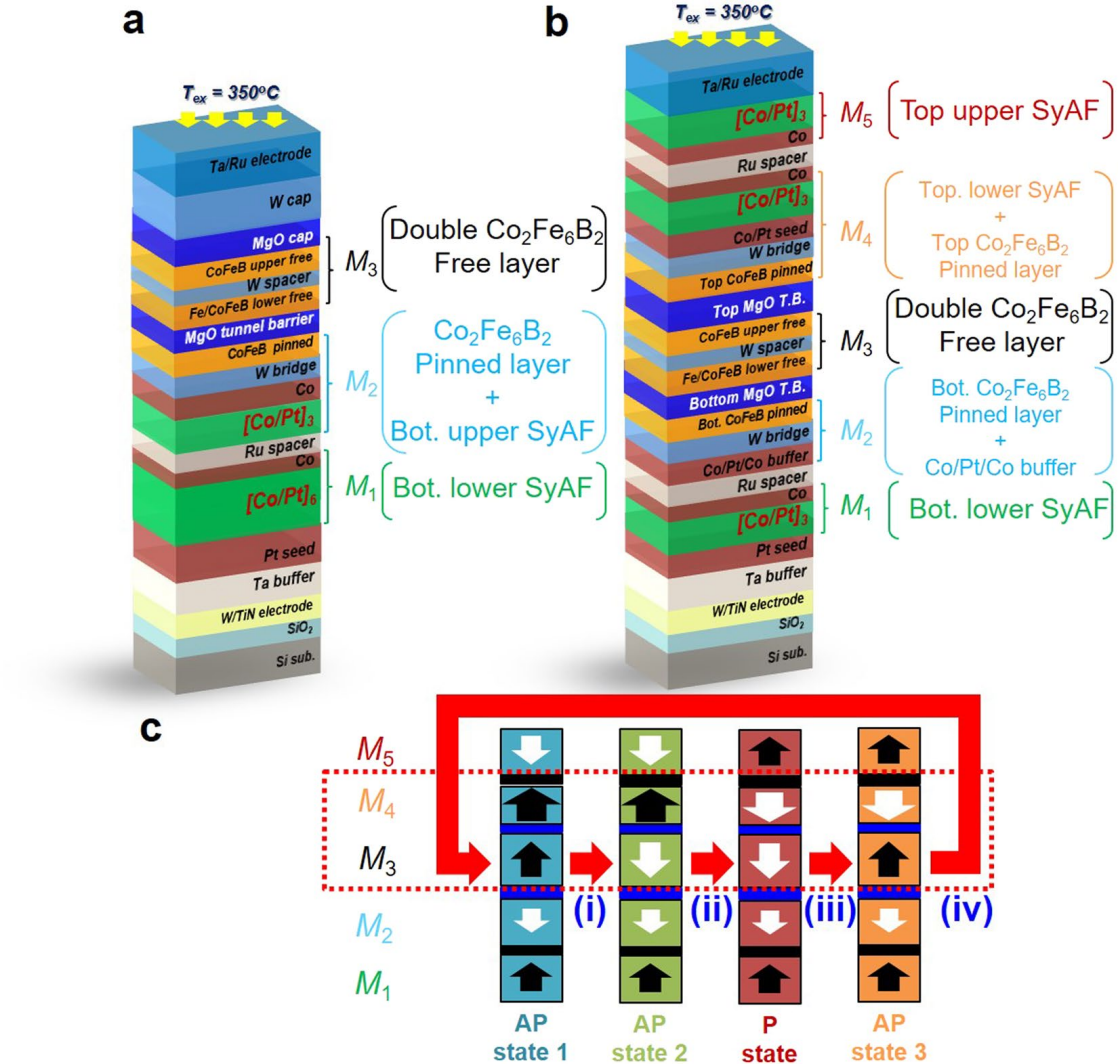
Schemes of p-MTJ spin-valves and design concept of the double pinned p-MTJ spin-valve. (**a**) Single pinned p-MTJ spin-valve with double free layer, (**b**) double-pinned p-MTJ spin-valve with double free layer, and (**c**) spin direction configuration of the double pinned p-MTJ spin-valve producing four-level resistance states.

The researches on p-STT MRAM have been based on improving three device parameters of the p-MTJ spin-valves^11–13^: the tunneling magnetoresistance (TMR) ratio greater than 150% for ensuring a memory margin, the thermal stability (Δ = *K*_*u*_V/*k*_*B*_T, where *K*_*u*_, V, *k*_*B*_, and T are the magnetic anisotropy energy, the volume of the free ferromagnetic layer, the Boltzmann constant, and the temperature, respectively) above 75 for a ten-year retention-time, and the switching current density of about 1 MA/cm^2^ for low power consumption. Moreover, these device parameters should be available at a back end of line (BEOL) temperature > 350 °C^16,17^. The conventional double MgO based p-MTJ spin-valve consist of upper and lower synthetic anti-ferromagnetic (SyAF) [Co/Pt]_n_ multilayer separated by a Ru spacer, a Co_2_Fe_6_B_2_ magnetic pinned layer, a MgO tunneling barrier, and Co_2_Fe_6_B_2_ magnetic free layers, as shown in Fig. 1a: called a single pinned p-MTJ spin-valve^18^. A single pinned p-MTJ spin-valve can generate only two resistance states: the high-resistance state (HRS) from the anti-parallel spin direction, and the low-resistance state (LRS) from the parallel spin direction between the Co_2_Fe_6_B_2_ free layer and the Co_2_Fe_6_B_2_ pinned layer. Thus, these two different resistance states have been used to operate only single-bit p-STT MRAM memory cells. However, the scaling down for terabit-level integration of the p-STT MRAM cells, to compete with current DRAM^19^, 3-dimensional (3D) NAND flash memory, and 3-dimensional cross-point memory, would be necessary to achieve the thermal stability required for 10-year retention time^20,21^ and multi-level memory-cell operation like that of current 3-D NAND flash memory^22,23^. In our research, we designed a double pinned p-STT-MTJ spin-valve exhibiting multi-level (i.e., four) resistance states. The double pinned p-STT MRAM was composed of three main ferro-magnetic component layers: the bottom pinned structure, double free-layer, and top pinned structure (see Fig. 1b). The TMR ratio was maximized by introducing a bottom single SyAF [Co/Pt]_n_ multilayers (Fig. 1b) because the upper [Co/Pt]_3_ SyAF multilayer anti-ferro-coupled with the lower [Co/Pt]_6_ SyAF multilayers (Fig. 1a) via a Ru spacer produced considerably high surface roughness^18^. The design concept of the double pinned p-STT MTJ will be explained in more detail in the following section. In addition, we investigated static magnetic properties of the double pinned p-MTJ spin-valve, tested the achievement of four-level magnetic-resistance states, and analyzed the operation mechanism of four-level resistances.

## Results

### Design of Double Pinned p-MTJ Spin-valve

The double pinned p-MTJ spin-valve was vertically stacked with the bottom electrode, bottom Co_2_Fe_6_B_2_ ferromagnetic pinned structure (called bottom pinned structure), double MgO based Co_2_Fe_6_B_2_ ferromagnetic free layer (called the double free-layer), top Co_2_Fe_6_B_2_ ferromagnetic pinned structure (called top pinned structure), and top electrode, as shown in Fig. 1b. The magnetic layers of the double pinned p-MTJ spin valve can be divided largely into five groups (*M*_1_, *M*_2_, *M*_3_, *M*_4_, and *M*_5_ layers). The bottom electrode was made by sputtering tungsten (W) and titanium nitride (TiN) layers on a thermally oxidized 300-mm-diameter Si wafer, followed by the chemical-mechanical-planarization (CMP). For the bottom pinned structure, the Ta buffer layer was used to intersect the f.c.c. crystalline texturing of the TiN electrode since the Ta layer had an amorphous structure. The face-centered-cubic crystalline Pt-seed-layer was used to form the L10 crystalline structure of the bottom-lower SyAF [Co (0.47 nm)/Pt(0.23 nm)]_3_ multilayer (*M*_1_ layer) so that its spin direction was perpendicularly upward, as shown in Fig. 1c^24–26^. These layers were anti-ferro-coupled with the single Co (0.51 nm)/ Pt(0.23 nm)/Co(0.47 nm) buffer layer via Ru spacer, called the single SyAF [Co/Pt]_n_ layer. Simultaneously, the single Co/Pt/Co buffer-layer was ferro-coupled to the bottom Co_2_Fe_6_B_2_ ferromagnetic pinned layer (0.95 nm) via the W bridge layer, defined as *M*_2_ layer. The spin direction of the *M*_2_ layer was perpendicularly downward, as shown Fig. 1c. Thus, the spin directions of the *M*_1_ and *M*_2_ layers always face vertically inward towards each other. In particular, the anisotropy exchange field (*H*_*ex*_) of the bottom pinned structure should be sufficiently higher than the coercivity (*H*_c_) of the top pinned structure to fix the spin direction of both the *M*_1_ and *M*_2_ layers. Then, the double Co_2_Fe_6_B_2_ free-layer (*M*_3_ layer) was stacked on the *M*_2_ layer, where the thicknesses of the bottom MgO tunneling-barrier, Fe insertion layer, lower Co_2_Fe_6_B_2_ ferromagnetic free layer, W spacer, upper Co_2_Fe_6_B_2_ ferromagnetic free layer, and top MgO tunneling-barrier layer were 1.15, 0.3, 1-0.05, 0.2, 1.05, and 1.0 nm, respectively. The spin direction of *M*_3_ layer was dependent of the polarity of the applied perpendicular-magnetic-field; i.e. vertically downward for a negative field and vertically upward for a positive field, as shown in Fig. 1c. In particular, the *H*_c_ of *M*_3_ layer should be considerably smaller than the *H*_c_ of the top pinned structure to make four different spin direction states between the *M*_3_ and *M*_4_ layers [i.e., anti-parallel (AP) state 1, AP state 2, parallel (P) state, and AP state 3]. For the top pinned structure, the Fe insertion layer (0.3 nm) and top Co_2_Fe_6_B_2_ ferromagnetic pinned layer (0.75 nm) were stacked on the *M*_3_ layer. The top-lower [Co(0.47 nm)/Pt(0.23 nm)]_3_ SyAF multilayer was ferro-coupled with the top Co_2_Fe_6_B_2_ ferro-magnetic pinned-layer (0.75 nm) via a W bridge layer and Co/Pt seed layer, which is defined as *M*_4_ layer. The spin direction of the *M*_4_ layer was dependent of the polarity of the applied magnetic-field. Simultaneously, *M*_4_ layer was always anti-ferro-coupled with the top-upper [Co(0.47 nm)/Pt(0.23 nm)]_3_ SyAF multilayers (*M*_5_ layer), via the Ru spacer. Thus, the spin direction of the *M*_5_ layer was always in the opposite of the *M*_4_ layers, as shown in Fig. 1(c). In particular, the number (**m**) of the top-lower [Co(0.47 nm)/Pt(0.23 nm)]_**m**_ SyAF multilayers should be higher than that (**n**) of the top-upper [Co(0.47 nm)/Pt(0.23 nm)]_**n**_ multilayers to produce four-different spin direction states between the *M*_3_ and *M*_4_ layers. If **m** is lower than **n**, only two different spin direction states would be generated between the *M*_3_ and *M*_4_ layers. Finally, a top Ta/Ru electrode was stacked on the *M*_5_ layer.

In summary, the design of the double pinned p-MTJ spin-valve could produce four-different spin direction states between the *M*_3_ and *M*_4_ layers: AP state 1 (perpendicularly upward spin direction for both *M*_3_ and *M*_4_ layers), AP state 2 (spin direction facing outward between *M*_3_ and *M*_4_ layers), P state (downward spin direction for both *M*_3_ and *M*_4_ layers), and AP state 3 (spin direction facing inward between the *M*_3_ and *M*_4_ layers, as shown in Fig. 1c). To form four-different spin direction states, we essentially need three key-design concepts: 1) for designing the bottom pinned structure, the spin direction of the *M*_1_ and *M*_2_ layers should face always inward toward each other, 2) for designing *M*_3_ layer, its *H*_c_ should be remarkably smaller than the *H*_c_ of the *M*_4_ layer to assure to produce four-different spin direction states between the *M*_3_ and *M*_4_ layers, and 3) for designing the top pinned structure, the *H*_*ex*_ of the *M*_4_ layers should be sufficiently higher than the *H*_c_ of the the *M*_3_ layer to avoid a write-error. These three key-design concepts will be treated later in detail.

### Design and Static Perpendicular-Magnetic Behaviour of Bottom Pinned Structure and Double Free-Layer

In the bottom pinned structure (*M*_1_ and *M*_2_ layers) in Fig. 1b, the spin direction of the *M*_2_ layer should always face perpendicularly inward toward with that of the *M*_1_ layer, as shown in Fig. 1c. Thus, this bottom pinned-structure needs as large *H*_*ex*_ of the *M*_2_ layer as possible. In our previous studies, the spin directions of the *M*_1_ and *M*_2_ layers are always facing perpendicularly inward against each other when the number of the bottom-upper SyAF [Co(0.4 nm)/Pt(0.2 nm)] layer (i.e., 3) is less than that of the bottom-lower SyAF [Co(0.4 nm)/Pt(0.2 nm)] layer (i.e., 6)^27–30^. Also, we implemented the p-MTJ spin-valves with a single SyAF [Co/Pt]_n_ layer which showed large *H*_*ex*_, as shown in Supplementary 1^18^.

**Figure 2:**
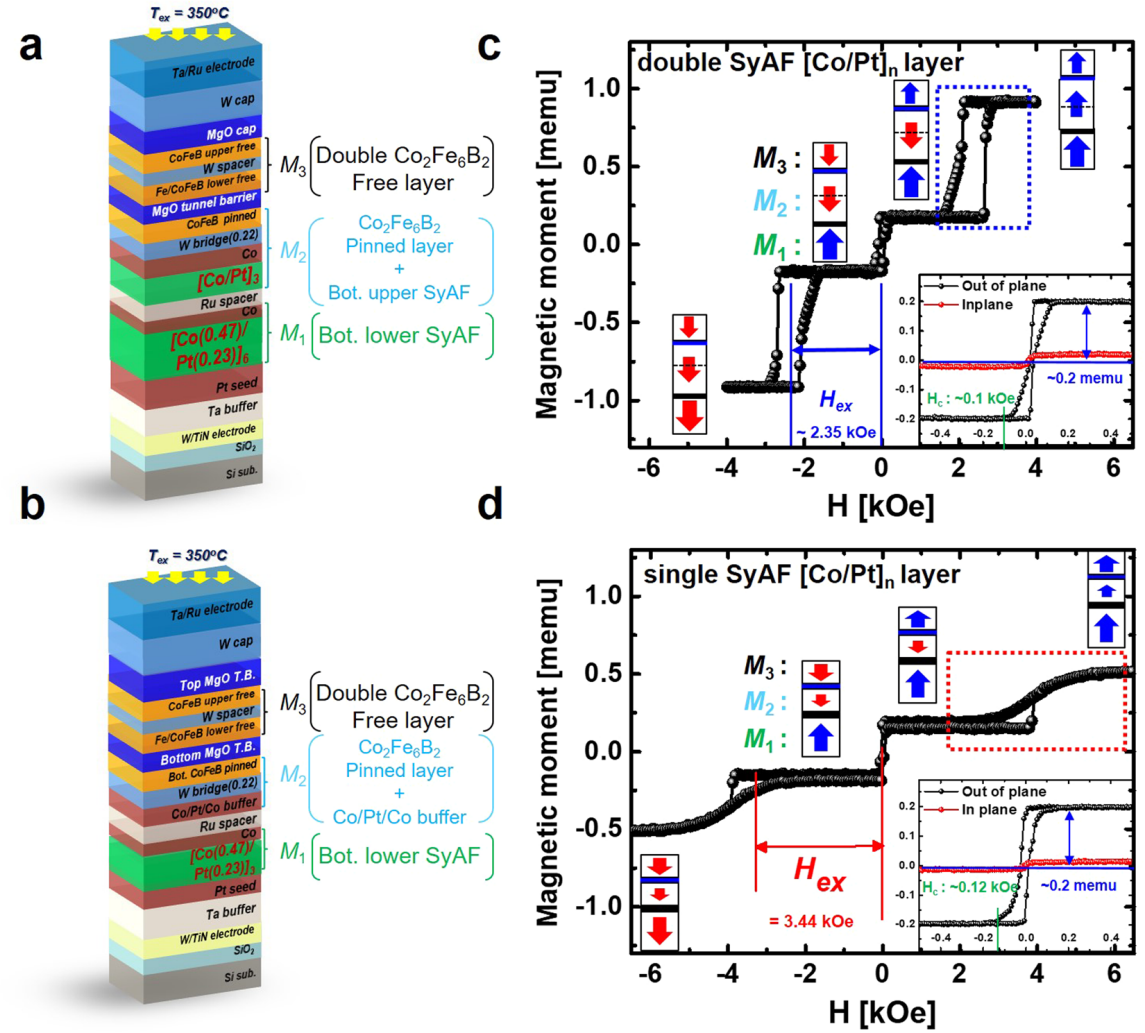
Dependence of static magnetic behavior (magnetic moments-vs.-applied magnetic field) of bottom pinned structure. Schemes of p-MTJ spin-valves with (**a**) double SyAF [Co/Pt]_n_ layer and (**b**) single SyAF [Co/Pt]_n_ layer. *M-H* curve of (**c**) double SyAF [Co/Pt]_n_ layer (**d**) a single SyAF [Co/Pt]_n_ layer.

Using this concept, we redesigned the bottom pinned p-MTJ spin-valve (Fig. 2a,b) with double and single SyAF [Co/Pt]_n_ layers to match that of the double pinned p-MTJ spin-valve and investigated its magnetic properties. The arrow magnitude and direction corresponded to the relative magnetic-moment and spin direction of the magnetic layers when the applied magnetic field is changed from +6.5 kOe to −6.5 kOe, as shown in Fig. 2c,d. The inset of Fig. 2c,d shows the magnetic properties of the Co_2_Fe_6_B_2_ free layers (*M*_3_ layers) of the p-MTJ spin-valve with double and single SyAF [Co/Pt]_n_ layers. The spin direction of both structures are aligned along the external field direction when external field is high in the upward direction (*H* > +5 kOe). When the external field becomes smaller than + 1.5 kOe, the spin direction of the *M*_2_ layers are switched opposite the external field as the field is not strong enough to overcome the anti-ferro coupling between *M*_1_ and *M*_2_ layers. The *M*_2_ layer squareness of the bottom p-MTJ spin-valve with single SyAF [Co/Pt]_n_ layers (red box of Fig. 2d) is degraded compared to that of the spin-valve with double SyAF [Co/Pt]_n_ layers (blue box of Fig. 2c). However, the *H*_ex_ is increased from 2.35 kOe to 3.44 kOe which would mean that the *M*_1_ and *M*_2_ layers of the p-MTJ spin-valve with single SyAF [Co/Pt]_n_ layers is more unsusceptible to switching. In addition, the peak-to-valley (Δ_P-V_) of the MgO tunneling barrier decreased from 2.03 nm to 1.75 nm when the thickness of the SyAF layers is reduced from 8.87 to 4.65 nm as shown in Supplementary 2. Note that the TMR ratio of the double pinned p-MTJ spin-valve is expected to increase by reducing the roughness of the MgO tunneling barrier^27–38^. Thus, the magnetic property of the double free-layer was not degraded; i.e., a good squareness and the magnetic moment of ~ 0.2 memu, as shown in the inset of Fig. 2d. This result indicates that a single SyAF [Co/Pt]_3_ multi-layers would be very suitable as a bottom pinned structure (*M*_1_ and *M*_2_ layers in Fig. 1b), since it could provide a sufficiently high *H*_*ex*_ of 3.44 kOe and would increase the TMR ratio because of a lower surface roughness of the MgO tunneling-barrier. The spin direction schematic in Fig. 2c,d represents the change only when the magnetic field is changed from +6.5k Oe to −6.5 kOe. A detailed magnetic switching behavior of the p-MTJ spin-valve with double and single SyAF [Co/Pt]_n_ layer under an external magnetic field sweep from −6.5 kOe to +6.5 kOe is shown in see Supplementary 3.

### Design and Static Perpendicular-Magnetic Behaviour of Top Co_2_Fe_6_B_2_ Ferro-Magnetic Pinned Structure

**Figure 3:**
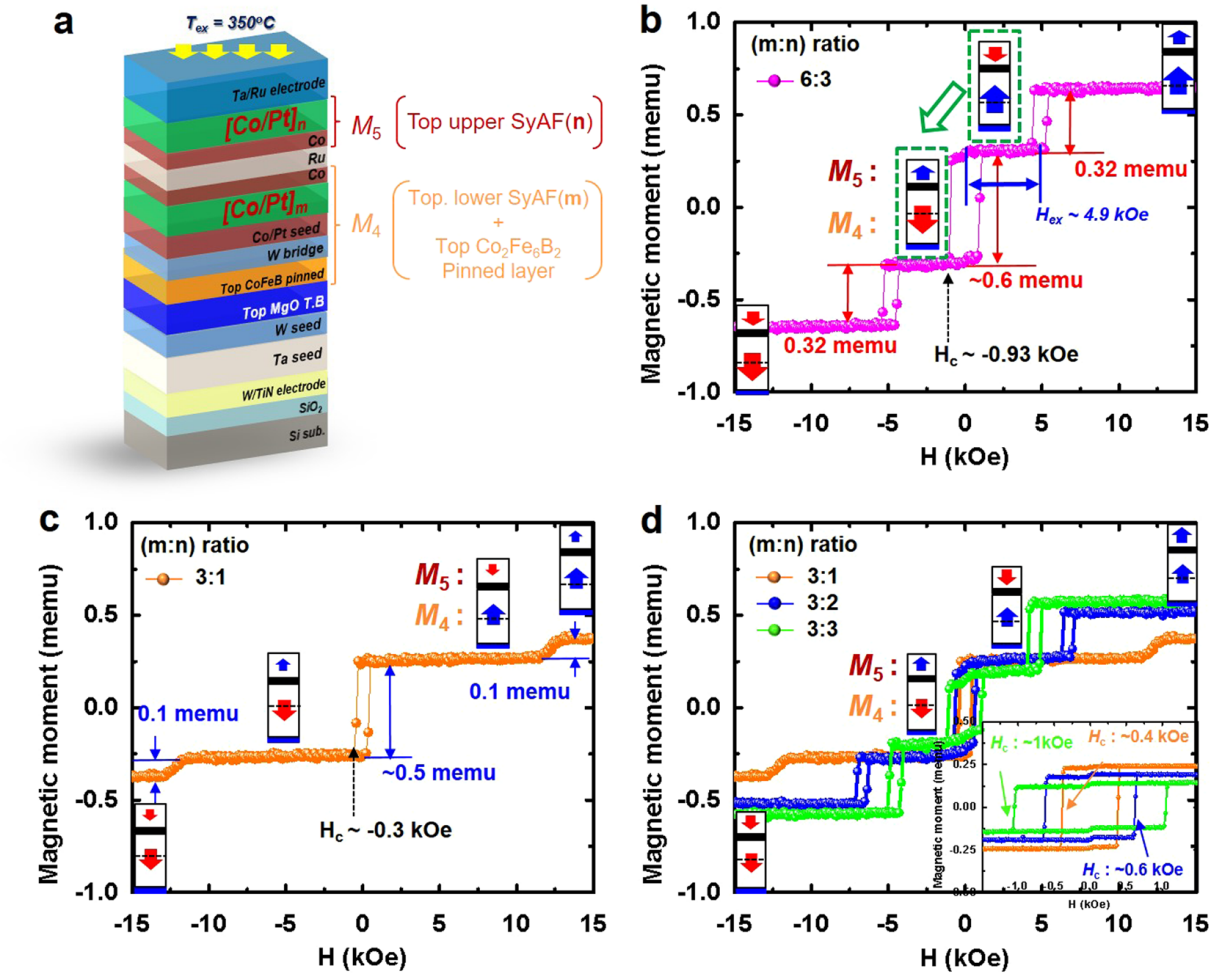
Dependence of static magnetic behaviour (magnetic moments-vs.-applied magnetic-field) of top pinned structure. (**a**) Scheme of top pinned structure design with [**m]** number of lower [Co/Pt] layers and [**n]** number of upper [Co/Pt] layers, (**b**) *M*-*H* loop of top pinned structure with **m**:**n** ratio of 6:3, (**c**) *M*-*H* loop of top pinned structure with **m**:**n** ratio of 3:1, and (**d**) *M*-*H* loops of top pinned structures with **m**:**n** ratios of 3:1. 3:2, and 3:3.

In the top pinned structure, the top Co_2_Fe_6_B_2_ magnetic pinned-layer was ferro-coupled with the top-lower SyAF [Co/Pt]_**m**_ layer via W bridge layer (*M*_4_ layer), which was then anti-ferro-coupled with the top-upper SyAF [Co/Pt]_**n**_ layer (*M*_5_ layer) via Ru spacer, as shown in Fig. 3a. In addition, the number of the top-lower [Co(0.47 nm)/Pt(0.23 nm)] layers **(m)** of the *M*_4_ layer should be less than the number of the top-upper [Co(0.47 nm)/Pt(0.23 nm)] layers **(n)** of the *M*_5_ layer, and the *H*_ex_ of the top pinned structure should be as large as possible to avoid a write-error. Unlike the bottom pinned structure in Fig. 2d, the top pinned structure should be able to generate four different electron-spin states between the *M*_3_ and *M*_4_ layer, resulting in four different resistance states. Thus, within the scanning magnetic-field range less than the *H*_ex_ of the top pinned structure, the spin direction of the *M*_4_ layer could be rotated from upward to downward or downward to upward when the polarity of the magnetic-field changes from positive to negative or from negative to positive. To test whether or not the spin directions of the top pinned structure were variable, we investigated the *M-H loop* of a basic top pinned-structure with the **m**:**n** ratio of 6:3 of the number of [Co(0.47 nm)/Pt(0.23 nm)] multilayers in the *M*_4_ and *M*_5_ layers, as shown in Fig. 3b. At the applied magnetic-field of +15 kOe, the spin direction of both the *M*_4_ and *M*_5_ layers faced perpendicularly upward. As the magnetic-field decreased from +15 to +4 kOe, the magnetic moment decreased from +0.6 to +0.3 memu, corresponding to the magnetic moment of the *M*_5_ layer (i.e., 0.3 memu) rotating the spin direction of the *M*_5_ layer from upward to downward. This occurred at the *H*_*ex*_ of ~4.9 kOe arising from the anti-ferro coupling across the Ru spacer layer. As the magnetic-field decreased from +4 to −4 kOe, the magnetic moment changed from +0.3 to −0.3memu, responding to the magnetic moment of the *M*_4_ and *M*_5_ layers (i.e., 0.6 memu), rotating the spin direction of the *M*_4_ layer from upward to downward. Simultaneously, the spin direction of the *M*_5_ layers rotated from downward to upward to hold the anti-ferro coupling via the Ru spacer stably. As a result, the spin directions of the *M*_4_ and *M*_5_ layers facing perpendicularly inward changed to facing perpendicularly outward. As the magnetic-field increased over −4 kOe, the spin direction of the *M*_5_ layer rotated from upward to downward so that the spin directions of both the *M*_4_ and *M*_5_ layers were perpendicularly downward. In contrast, as the magnetic-field changed from negative to positive direction, the change of the spin directions of the *M*_4_ and *M*_5_ layers followed the same order as the magnetic-field changed from positive to negative direction. In particular, the spin directions of the *M*_4_ and *M*_5_ layers facing perpendicularly outward changed to facing perpendicularly inward. Thus, this top pinned structure could produce two spin directions between the *M*_4_ and *M*_5_ layers when the magnetic-field is greater than the *H*_*c*_ of the top pinned structure; facing perpendicularly outward for the negative magnetic-field and facing perpendicularly inward for the positive magnetic-field. Although the top-lower SyAF [Co(0.47 nm)/Pt(0.23 nm)]_6_ layer and top-upper SyAF [Co(0.47 nm)/Pt(0.23 nm)]_3_ layer and could provide variable spin directions between the *M*_4_ and *M*_5_ layers, the top-lower SyAF [Co(0.47 nm)/Pt(0.23 nm)]_6_ layer is too thick to maximize the TMR ratio of the p-MTJ spin-valve. The TMR ratio is strongly dependent on the coherent tunneling of the Δ_1_ Bloch state induced from the hybridization of the Fe-d_z_^2^ and O-p_z_ orbitals at the MgO/CoFeB interface. Even a small defect at the interface reduces the coherent tunneling of the spin-polarized electrons^31,39^. The roughness increases with the number of top-lower SyAF [Co/Pt]_n_ layers from about 150 pm to 240 pm as seen in Supplementary 4. The roughness needs to be reduced to maximize the TMR ratio to assure four levels of the double pinned p-MTJ spin-valve.

To reduce the thickness of the top pinned structure, first of all, we investigated the *M-H loop* of the top pinned structure with the ratio of the [Co(0.47 nm)/Pt(0.23 nm)]_3_ layers of the *M*_4_ and *M*_5_ layers (**m:n** ratio) of 3:1, as shown in Fig. 3c. Its static magnetic behaviour was similar to the top pinned structure with the ratio of **m:n** ratio of 6:3, showing the magnetic moment of the *M*_4_ and *M*_5_ layers, *H*_c_, and *H*_ex_ were 0.1 and 0.5memu, ~0.3 kOe, and ~12 kOe. Although this top pinned structure could produce two variable spin directions between the *M*_4_ and *M*_5_ layers, the *H*_c_ of ~0.3 kOe was too small to generate four different spin direction states between the *M*_3_ and *M*_4_ layers in the double pinned p-MTJ spin-valve (Fig. 1c) since the *H*_c_ of the top pinned structure (i.e., ~0.3 kOe) was not sufficiently higher than the *H*_c_ of the double free-layer (Fig. 2d: i.e., ~0.2 kOe) to avoid the write error. Thus, we observed the dependency of the *H*_c_ of the top pinned structure on the **m:n** ratio, as shown in Fig. 3d. The **m:n** ratios of 1:3, 2:3, and 3:3 showed *H*_c_ values of ~0.4, ~0.6, and ~1 kOe, as shown in the inset of Fig. 3d, and all **m:n** ratios demonstrated two variable spin directions between the *M*_4_ and *M*_5_ layers. This result indicates that the *H*_c_ of the top pinned structure increases with the number of [Co(0.47 nm)/Pt(0.23 nm)]_3_ layers in the *M*_5_ layer, while *H*_ex_ decreases with the number of [Co(0.47 nm)/Pt(0.23 nm)]_3_ layers in the *M*_5_ layer. In particular, the **m:n** ratio of 3:3 showed a sufficient *H*_c_ (i.e., ~1 kOe), which could produce four different spin direction states between the *M*_3_ and *M*_4_ layers (Fig. 1c) since it is considerably larger than the *H*_c_ of the double free-layer (~0.1 kOe) (Fig. 2d). Recall that at the **m:n** ratio of 3:3 the static magnetic moment of the *M*_4_ layer was slightly larger than that of the *M*_5_ layer so that the spin direction of the top pinned structure (*M*_4_ and *M*_5_ layers) faced vertically outward for the negative applied magnetic-field and vertically inward for the positive applied magnetic-field. If **n** is larger than **m**, the spin directions of the *M*_4_ and *M*_5_ layers could not be variable. Therefore, the design of choosing a proper *H*_c_ and *H*_ex_ of the top pinned structure would be a key research to stably produce four different spin direction states between *M*_3_ and *M*_4_ layers.

### Static Perpendicular-Magnetic Behaviour and Multi-level TMR ratio for Double Pinned p-MTJ Spin-Valve

**Figure 4:**
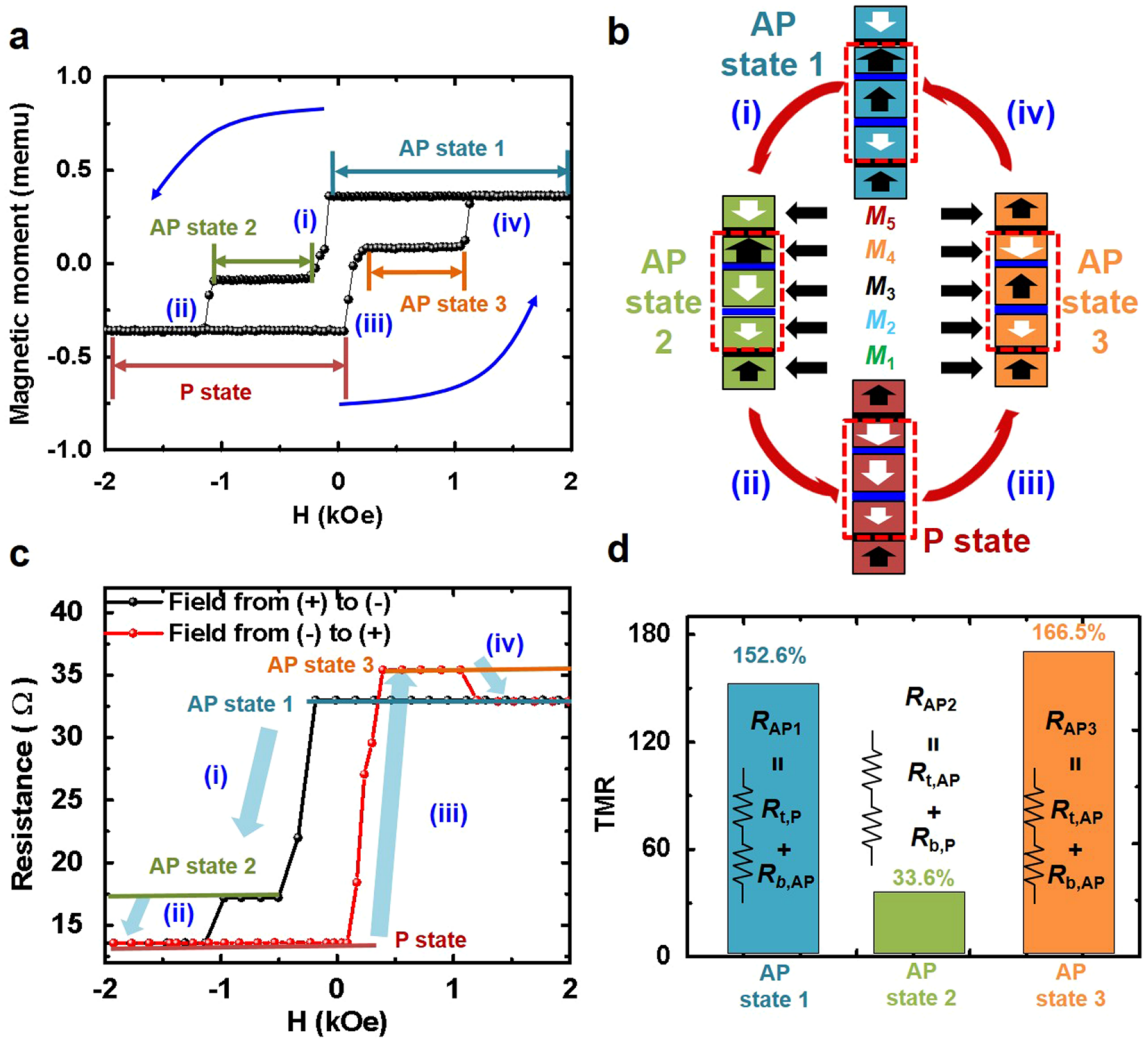
Magnetic and resistance properties of double pinned p-MTJ spin-valve in narrow scanning magnetic-field range (−2 kOe to +2 kOe). (**a**) *M*-*H* loop, (**b**) four-different spin-electron-directions depending on the polarity and magnitude of the scanning magnetic-field, (**c**) *R*-*H* loop of double pinned p-MTJ spin-valve with cell size of 2 μm^2^, and (**d**) TMR ratios of double pinned p-MTJ spin-valve.

By combining the top (3:3 of **m:n** in Fig. 3d) and bottom p-MTJ structures with the double free-layer (Fig. 2d), we fabricated the double pinned p-MTJ spin-valve shown in Fig. 1b. The *M-H* loop of the double pinned p-MTJ spin-valve showed the *H*_ex_ of 4.9 kOe when the applied magnetic-field was scanned from −6.5 kOe to +6.5 kOe, as shown in Supplementary 5a. In addition, the resistance-vs.-magnetic-field (*R-H*) loop presented only three resistance states, when the applied magnetic-field was scanned from −*H*_ex_ to +*H*_ex_ kOe, as shown in Supplementary 5b,c. In order to produce four different resistance states, thus, the maximum scanning range of the applied magnetic-field should be sufficiently less than $\pm$*H*_ex_ (i.e., ~4.2 kOe) of the bottom pinned p-MTJ spin-valve, but greater than ±*H*_ex_ (i.e., ~1 kOe) of the top pinned structure (*M*_4_ and *M*_5_ layers); i.e., $\pm$ 2 kOe. Thus, four different spin directions between the *M*_3_ and *M*_4_ layers could be stably produced when the applied magnetic-field was scanned from −2 kOe to +2kOe, as shown in the *M-H* loop of Fig. 4a. First, the AP state 1 was produced when the applied magnetic-field was scanned from +2 kOe to +0.5 kOe, where the spin directions of both the *M*_3_ and *M*_4_ layers were vertically upward and parallel while the spin directions of the *M*_4_ and *M*_5_ layers faced vertically inward toward each other via an anti-ferro-coupling, as shown Fig. 4a,b. Recall that this result corresponds to the combination of the top pinned structure (*M*_4_ and *M*_5_ layers) in Fig. 3d and the bottom pinned structure (*M*_1_ and *M*_2_ layers) with the double free-layer (*M*_3_ layer) in Fig. 2d at the positive applied magnetic-field. Then, when the applied magnetic-field was scanned from +0.5 kOe to −0.5 kOe, the spin direction of only the *M*_3_ layer was rotated from upward to downward while the spin directions of both the *M*_4_ and *M*_5_ layer did not change, generating the AP state 2, where the spin direction of the *M*_3_ layer faced vertically outward against that of the *M*_4_ layer, as shown in **i** in Fig. 4a,b. Furthermore, when the applied magnetic-field was scanned from −0.5 kOe to −2.0 kOe, the spin directions between the *M*_4_ and *M*_5_ layers facing vertically inward were rotated to face vertically outward while the spin direction of the double free-layer was sustained downward, forming the P state, where the spin directions of both *M*_3_ and *M*_4_ layers were vertically downward and in parallel, as shown in **ii** in Fig. 4a,b. Subsequently, when the applied magnetic-field was scanned from −2.0 kOe to +0.5 kOe, the spin direction of only the *M*_3_ layer rotated from downward to upward while the spin directions of both the *M*_4_ and *M*_5_ layers remained facing vertically outward from each other, generating the AP state 3, where the spin direction of *M*_3_ layer faced vertically inward against that of the *M*_4_ layer, as shown in **iii** in Fig. 4a,b. Finally, the spin directions between the *M*_4_ and *M*_5_ layers facing vertically outward were rotated to face vertically inward while the spin direction of the *M*_3_ layer remained facing upward, returning to the AP 1 state, where the spin directions of both the *M*_3_ and *M*_4_ layers facing vertically inward transited to facing vertically and parallel upward, as shown in **iv** in Fig. 4a,b. As a result, four different spin directions between the *M*_3_ and *M*_4_ layers could be stably produced in a magnetic-field scanning range of $\pm$2.0 kOe.

The *R-H* loop corresponding to the *M-H* loop in Fig. 4a was shown in Fig. 4c. The resistance changed from the AP state 1, AP state 2, AP state 3, P state, and AP state 1 when the applied magnetic-field was scanned from +2.0 kOe, −2.0 kOe, and +2.0 kOe. The sequence of a higher resistance of the double pinned p-MTJ spin-valve was followed by AP state 3, AP state 1, AP state 2, and P state. The highest resistance was achieved when the *M*_3_ layers became the anti-parallel states against both the *M*_2_ and *M*_4_ layers; i.e., the AP state 3, corresponding to the sum of serial connection (*R*_AP3_) of the anti-parallel resistance between the *M*_3_ and *M*_4_ layers (*R*_*t*,AP_) with the anti-parallel resistance between the *M*_2_ and *M*_3_ layers (*R*_*b*,AP_), as shown in Fig. 4d. The second highest resistance was obtained, when the *M*_3_ layers became the parallel state against the *M*_4_ layers while it was in the anti-parallel state against the *M*_2_ layers; i.e., the AP state 1, responding to the sum of serial connection (*R*_AP1_) of the parallel resistance between the *M*_3_ and *M*_4_ layers (*R*_*t*,P_) with the anti-parallel resistance between the *M*_2_ and *M*_3_ layers (*R*_*b*,AP_). The third highest resistance was achieved, when the *M*_3_ layers became the anti-parallel state against the *M*_4_ layers while it did the parallel state against the *M*_2_ layers; i.e., the AP state 2, indicating to the sum of serial connection (*R*_AP2_) of the anti-parallel resistance between the *M*_3_ and *M*_4_ layers (*R*_*t,A*P_) with the parallel resistance between the *M*_2_ and *M*_3_ layers (*R*_*b*,P_). Note that the anti-parallel resistance between the *M*_2_ and *M*_3_ layers (*R*_*b*,AP_) is much larger than the anti-parallel resistance between the *M*_3_ and *M*_4_ layers (*R*_*t,A*P_) since the thickness of the bottom MgO tunneling barrier (1.15 nm) was greater than that of the top MgO tunneling barrier (1.0 nm). This is confirmed by the high-resolution transmission-electro-microscopy (HR-TEM) observation shown in Supplementary 3. Thus, the resistance of the AP state 1 was larger than that of the AP state 2.

Multi-level TMR ratios of the double pinned p-MTJ spin-valve were measure by CIPT (current-in-plane tunneling) measurement scanning the magnetic-field of $\pm$2 kOe. The TMR ratios were 152.6% for AP state 1, 33.6% for AP state 2, and 166.5% for AP state 3. These values correlated well with the four different resistance levels in the *R-H* loop in Fig. 4c. A higher TMR ratio was accounted for when the spin direction of the *M*_3_ layer becomes anti-parallel to that of the *M*_2_ layer (i.e., *R*_*b*,AP_). This result evidently indicates that the double pinned p-MTJ-spin-valve can perform multi-level (i.e., four-level) non-volatile memory-cell operation.

## Discussion

Our proposed double pinned p-MTJ spin-valve well demonstrated four-level resistance as a multi-level p-STT MRAM-cell, resulting in the TMR ratios of 152.6, 33.6, and 166.5%. The maximum TMR ratio of the double pinned p-MTJ spin-valve (166.5%) was slightly less than that of a single pinned p-MTJ spin-valve (i.e., 180%), since the Pt atoms in the top pinned structure diffused into both top and bottom MgO tunneling barrier so that the coherent tunneling of the spin-electron would be decreased as shown Supplementary 6. Thus, research on how to avoid the Pt diffusion from the top pinned structure is necessary; i.e., research on the design of a nano-scale buffer layer preventing Pt atom diffusion. In addition, to minimize a write-error originated between four-level resistances, the differences between four-level resistances should be as constant as possible. Thus, research on choosing a proper thickness between the top and bottom MgO tunneling barriers is also necessary. Success in the above-mentioned research will enable us to fabricate a terabit-level p-STT MRAM for embedded, stand-alone, and neuromorphic devices.

## Methods

The p-MTJ spin-valves were fabricated using a 12-inch-wafer multi-chamber cluster-magnetron sputtering-system under a high vacuum (less than 1 × 10^−8^ torr). In particular, the conventional p-MTJ spin-valve with the top double free-layer and double SyAF [Co/Pt]_n_ layers in Fig. 1a were fabricated by vertically stacking a 12-inch SiO_2_ wafer, W/TiN bottom electrode, Ta buffer layer, Pt seed layer, bottom-lower SyAF [Co(0.47 nm)/Pt(0.23 nm)]_6_ layers/Co(0.51 nm) (*M*_1_ layer), Ru spacer layer (0.85 nm), Co(0.51 nm)/Pt(0.23 nm)/ bottom-upper SyAF [Co(0.47 nm)/Pt(0.23 nm)]_3_ layers, and a Co buffer layer (0.47 nm). The W bridge layer of 0.22 nm was used to ferro-couple the bottom-upper SyAF layer to the pinned layer. The p-MTJ consisted of a Co_2_Fe_6_B_2_ bottom pinned layer (1.05 nm), MgO tunneling barrier (1.15 nm), Fe insertion layer (0.3 nm), Co_2_Fe_6_B_2_ free layers (1.05 nm), W spacer layer (0.4 nm), Co_2_Fe_6_B_2_ (1.05 nm), and MgO (1.0 nm)/W capping layer. The ferro-coupled bottom-upper SyAF [Co(0.47 nm)/Pt(0.23 nm)]_3_ layers and the Co_2_Fe_6_B_2_ bottom pinned layer is defined as the *M*_2_ layer. The bottom pinned structure of the double pinned p-MTJ spin-valve using a single SyAF [Co/Pt]_n_ layer and double free-layer were fabricated wherein the ratio of the number of [Co/Pt]_n_ layers between the upper and lower SyAF [Co/Pt]_n_ layer was varied from 3:6 (i.e., a double SyAF [Co/Pt]_n_ layer) to 0:3, as shown in Fig. 1b. In addition, a Co/Pt/Co buffer layer was used to bridge instead of the top-upper SyAF [Co(0.47 nm)/Pt(0.23 nm)]_3_ layer (compare Fig. 1a,b). The MgO capping layer of the conventional double MgO-based p-MTJ spin-valve structure was used as the top MgO tunneling barrier followed by an Fe insertion layer (0.3 nm), Co_2_Fe_6_B_2_ (0.75 nm) top pinned layer, W bridge layer (0.42 nm), Co(0.47 nm)/Pt(2 nm) buffer layer, and top-lower SyAF [Co(0.47 nm)/Pt(0.23 nm)]_3_ layer/ Co(0.51 nm). The ferro-coupled Co_2_Fe_6_B_2_ top pinned layer and the top-lower SyAF [Co(0.47 nm)/Pt(0.23 nm)]_3_ layer is defined as the top-pinned layer (*M*_4_ layer). Following the *M*_4_ layer is the Ru spacer layer (0.85 nm), Co(0.51 nm)/Pt(0.23 nm)/top-upper SyAF [Co(0.47 nm)/Pt(0.23 nm)]_3_ layer (*M*_5_ layer), and Ta/Ru top electrode. The spin-valves were *ex-situ* annealed at 350 °C for 30 min under a vacuum below 10^−6^ torr and a perpendicular magnetic-field of 3 tesla. The TMR ratios of the double pinned p-MTJ spin-valves fabricated on 12-inch Si wafers were estimated by using CIPT at room temperature. The wafers were cut into 1 × 1 cm^2^ pieces. The magnetic properties of the double pinned spin-valves were characterized by using vibrating-sample magnetometer (VSM) at room temperature. The *R-H* curve was measured with a p-MTJ spin-valve with the cell size of 2-μm × 2-μm. The 2-μm-scale p-MTJ spin-valves were wire-bonded to the sample holder and were installed into a home-made electrical probing system with a ~1 Tesla electromagnet using a Keithley 236 source measure unit and Agilent B2902A semiconductor parameter analyzer.

## Supplementary information

Supplementary Info
