# Supplementary material for: Double Pinned Perpendicular-Magnetic-Tunnel-Junction Spin-Valve Providing Multi-level Resistance States
Source: Sci Rep. 2019 Aug 15;9:11932. doi: 10.1038/s41598-019-48311-0 (PMC6695488; doi:10.1038/s41598-019-48311-0)
Supplement: Supplementary file 1 — Supplementary Info [file 41598_2019_48311_MOESM1_ESM.pdf]

Double Pinned Perpendicular-Magnetic-Tunnel-Junction Spin-Valve Providing Multi-level Resistance States

Jin-Young Choi[1], Han-Sol Jun[2], Kei Ashiba [1,3], Jong-Ung Baek[2], Tae-Hun Shim[1] & Jea-Gun Park[*1,2]

[1]*MRAM Center, Department of Electronics and Computer Engineering, Hanyang University, Seoul, 04763, Republic of Korea.*
[2]*MRAM Center, Department of Nanoscale Semiconductor Engineering, Hanyang University, Seoul, 04763, Republic of Korea.*
[3]*Wafer Engineering Department, SUMCO CORPORATION, 1-52 Kubara, Imari, Saga, 849-4256 Japan.*

*Corresponding author:
Jea-Gun Park
17 Haengdang-dong, Seongdong-gu, Seoul 04763, Republic of Korea
Tel. (+82)-2-2220-0234; fax (+82)-2-2296-1179.
E-mail address: parkjgl@hanyang.ac.kr

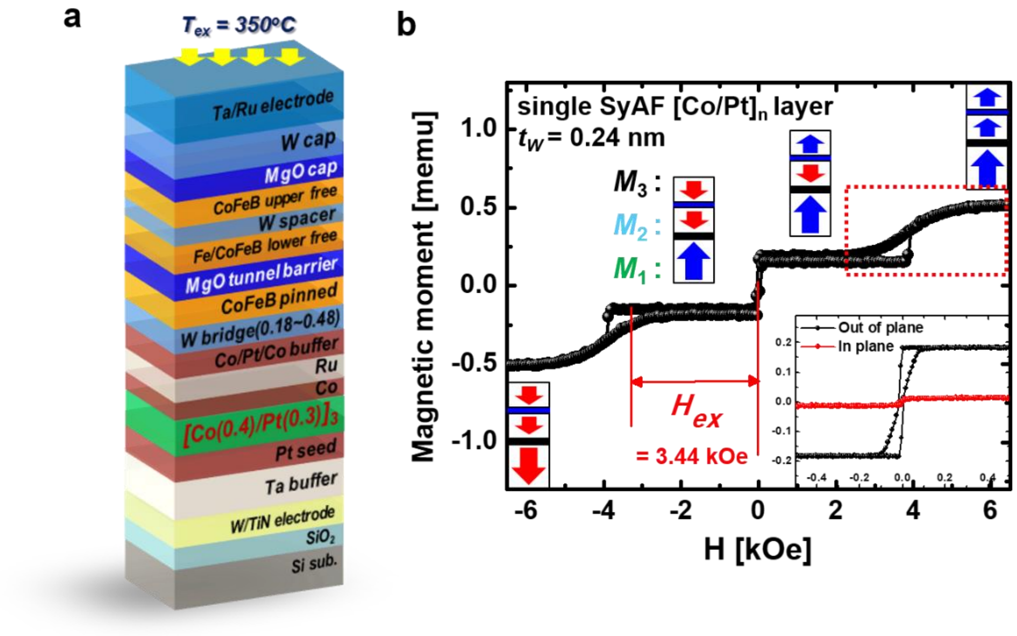

Supplementary 1. Scheme of the conventional single $[Co/Pt]_n$ SyAF p-MTJ spin-valve with double free layer. (a) detailed structure and (b) *M*-*H* loop in wide scanning magnetic-_field range (−6.5 kOe to +6.5 kOe) of single pinned p-MTJ spin-valve. [Sci. Rep. 8 (2018) 4–10]

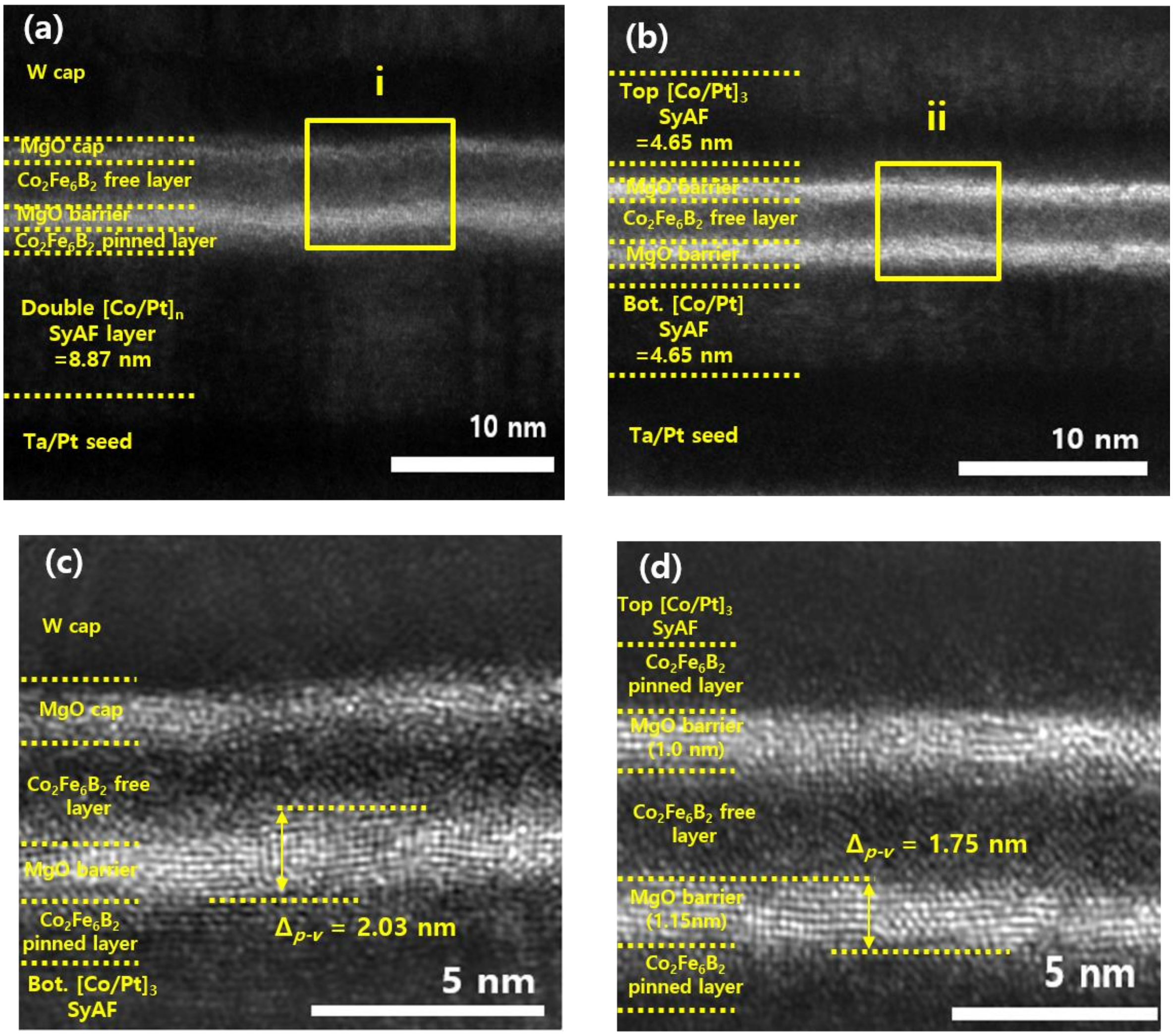

Supplementary 2. Crystallinity of MgO tunneling barrier and capping layer of (a) conventional p-MTJ spin-valve with double SyAF $[Co/Pt]_n$ layer and (b) double pinned p-MTJ spin-valve with single SyAF $[Co/Pt]_n$ layer, high resolution image obtained from (c) inset i of Supplementary 2a and (d) inset ii of Supplementary 2b

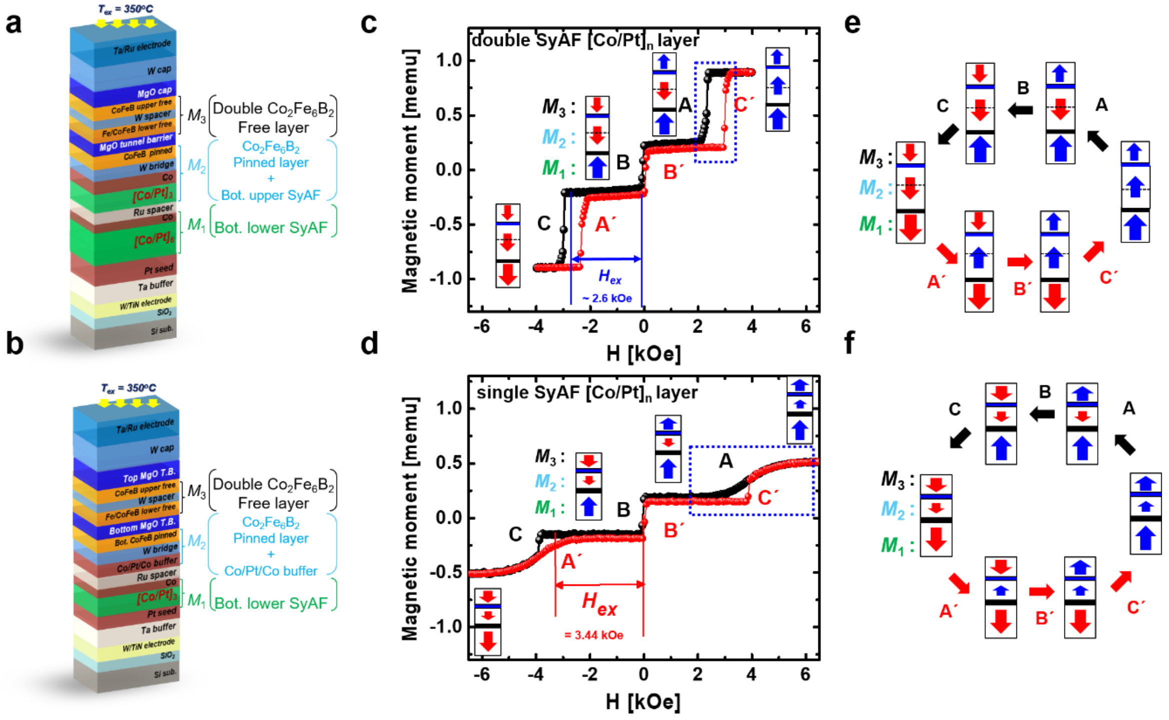

Supplementary 3. Switching mechanism of the single pinned p-MTJ spin-valve with double and single SyAF [Co/Pt]n layer. Schematic of single pinned p-MTJ spin-valve structure with (a) double SyAF $[Co/Pt]_n$ layer and (b) single SyAF $[Co/Pt]_n$ layer. M-H loop of single pinned p-MTJ spin-valve with (c) double SyAF $[Co/Pt]_n$ layer and (d) single SyAF $[Co/Pt]_n$ layer. The corresponding switching behavior of the magnetic layers (M1, M2, and M3) of single pinned p-MTJ spin-valve with (e) double SyAF $[Co/Pt]_n$ layer, and (f) single SyAF $[Co/Pt]_n$ layer

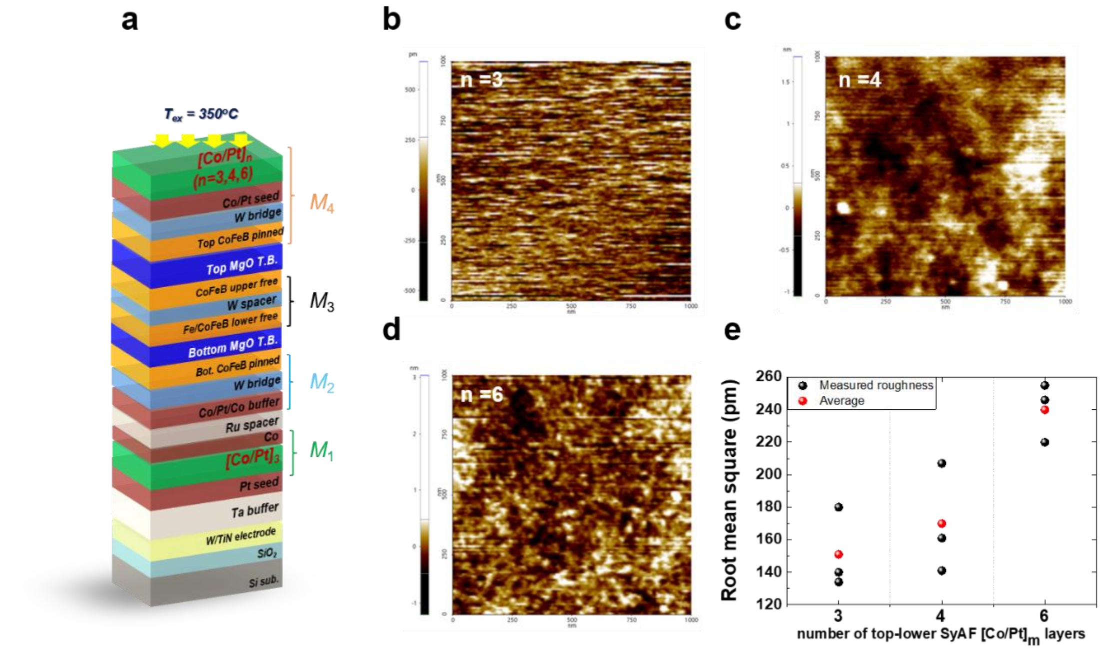

Supplementary 4. Surface roughness of double pinned p-MTJ structure depending on number of top upper $[Co/Pt]_m$ SyAF layers. (a) Schematic of double pinned p-MTJ structure. AFM image of (b) m=3 (c) m=4 and (d) m=6. (e) Root mean squared depending on number of top upper $[Co/Pt]_m$ SyAF layers.

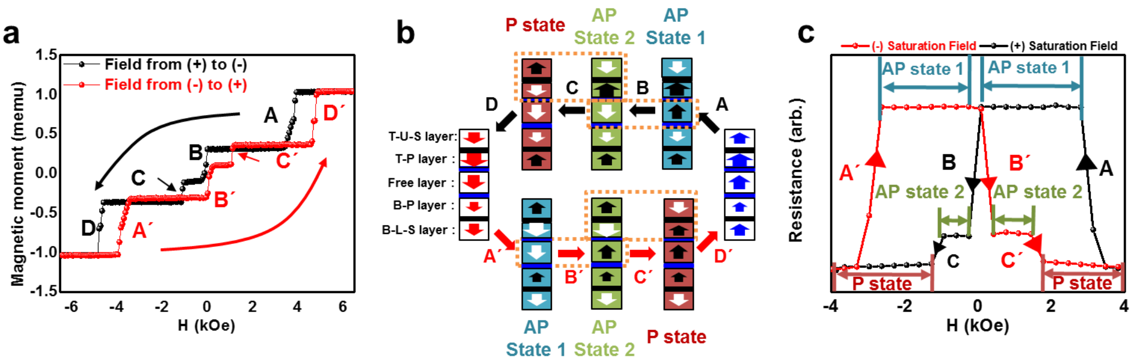

Supplementary 5. Magnetic and resistance properties of double pinned p-MTJ spin-valve in wide scanning magnetic-field range (−6kOe to +6 kOe). (a) *M-H* loop, (b) three different electron-spin directions depending on the polarity and magnitude of the scanning magnetic-_field, (c) *R-H* loop showing three resistance states.

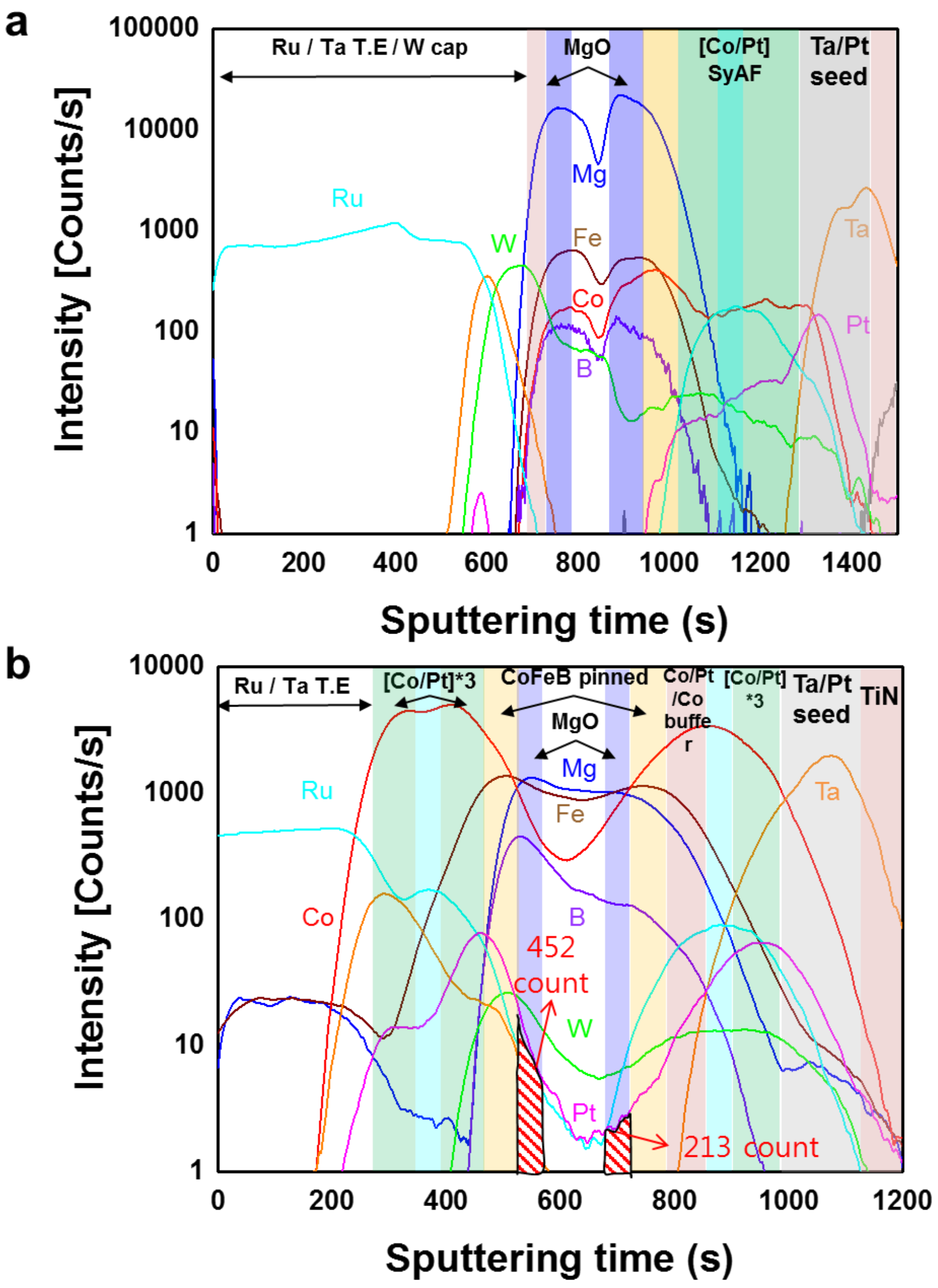

Supplementary 6. Cross-sectional chemical composition measured by SIMS of (a) conventional single pinned p-MTJ spin-valve showing no Pt atom diffusion into the MgO tunneling barrier, and (b) double pinned p-MTJ spin-valve showing Pt atom diffusion into both top and bottom MgO tunneling barriers.
